# Review of health and non-health sector indicators for monitoring service provision along the continuum of care for maternal health

**DOI:** 10.1186/s13104-020-04984-9

**Published:** 2020-03-13

**Authors:** Mamothena Carol Mothupi, Lucia Knight, Hanani Tabana

**Affiliations:** University of the Western Cape School of Public Health, Robert Sobukwe Rd, Bellville, Cape Town, 7535 South Africa

**Keywords:** Continuum of care for maternal health, Health service indicators, Social determinants of health indicators, Adequacy construct for the continuum of care for maternal health

## Abstract

**Objective:**

This study uses health and non-health sector data sources to select and assess available indicators for service provision along the continuum of care for maternal health at subnational levels in South Africa. It applies the adequacy approach established in another study to assess the multi-dimensionality of available indicators. Using adequacy and the process of assessment in the study, the comprehensiveness of the continuum of care for improving maternal health outcomes can be assessed.

**Results:**

We found 27 indicators of care utilization and access, linkages of care, and quality of care from the routine district health information system. The General Household Survey contained 11 indicators for the social determinants of health on the continuum of care framework. Indicator gaps include health promotion during and after pregnancy, maternal nutrition, empowerment and quality of care. At present, the available indicators measure about 74% of the interventions on the continuum of care framework. We make recommendations regarding improvements needed to better measure and monitor the continuum of care for maternal health. These involve actions within the health system and include integration of non-health system indicators.

## Introduction

**Fig. 1:**
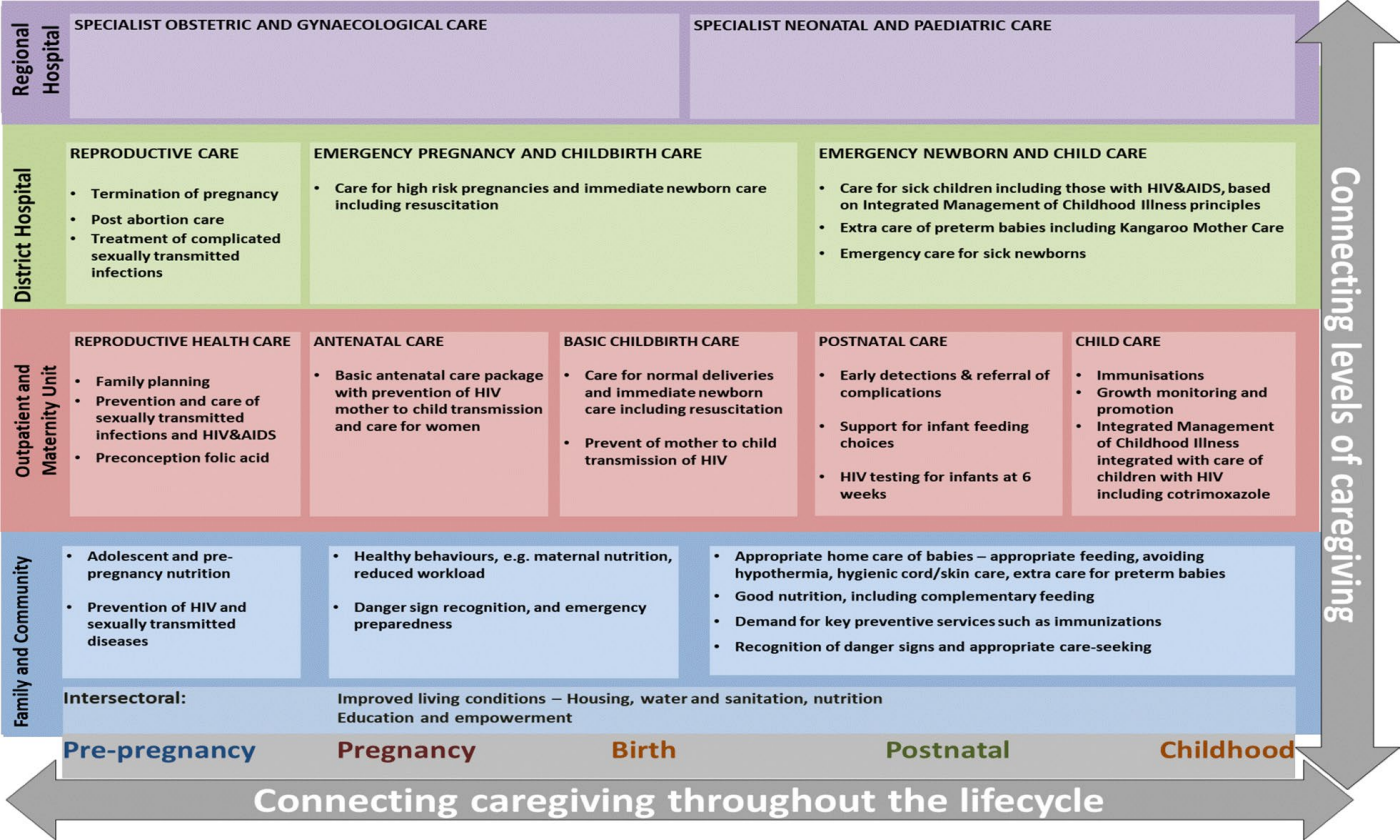
Continuum of care framework for maternal and newborn health in South Africa, [2]. The continuum of care framework for South Africa was developed by national health system stakeholders and decision makers [2]. It outlines important evidence-based interventions to improve maternal and child health outcomes across the continuum of care. Our study focuses on maternal health aspects. Interventions are implemented across the lifecycle from pre-pregnancy to postnatal period. The levels of care are outlined vertically, and the framework recommends connectedness or linkages between these levels to improve care. In addition, connection between intervention packages (boxed and colour coded) are important. As a primary health care framework, delivery of services on the continuum of care occurs at the district level and below. Besides health system interventions are “intersectoral factors” that represent important social determinants of health for maternal health. These include good living conditions, empowerment and education

The continuum of care is a strategy for improving the efficiency and effectiveness of service delivery for maternal health [1, 2]. It is the delivery of services from preconception to the postnatal period, including those related to social determinants of health. The continuum of care (CoC) framework, developed by national stakeholders in South Africa, is presented in Fig. 1. It outlines linked intervention packages from the family/community to the district level of care. South Africa (SA) has a strategic goal to deliver and monitor services along the CoC in maternal and related health areas [3, 4]. However, there is a gap in defining the indicator set for monitoring service delivery (mainly inputs, outputs and processes) along the CoC to support these goals.

In a previous study, we described the construct of adequacy, which emerged from a systematic review and critical interpretive synthesis of gaps in measurement of the CoC [5]. The adequacy approach states that the CoC should be measured and monitored in a comprehensive and multidimensional manner. This means all aspects of timely access to care, quality of care, linkages between levels of care, and social determinants of health should be measured. The framework in Fig. 1 guides the essential interventions and highlights their linkages, while the adequacy approach integrates multidimensional quality of care measurement.. In this study we used the framework in Fig. 1 and the adequacy construct to (i) propose an indicator tool for the CoC for maternal health in SA, and (ii) describe current gaps to be addressed in improving monitoring and provision of services.

## Main text

### Methods

In this study we assess available indicators currently used for health and non-health sector policy and planning in government programs. They thus have a defined monitoring purpose which is re-assessed for suitability to the CoC framework.

#### Indicator extraction

We used the routine district health information system (DHIS) to extract relevant health system indicators. The DHIS monitors health programmes, track patients and map service availability in the health system in SA [6]. The National Indicator Data Set (NIDS) within the DHIS contains indicators of service inputs, processes, outputs and outcomes (where relevant) extracted for this study, for the reference period April 2017–March 2019 [7]. For social determinants of health/intersectoral factors as outlined in Fig. 1, we assessed datasets on the Statistics SA Nesstar portal and selected the General Household Survey (GHS) (reference year 2017) as the most suitable source. The GHS is annually collected and contains data on all intersectoral factors, which are used in policy and planning in SA [8, 9]. All GHS data can be obtained from the DataFirst Portal of the University of Cape Town in SA [10].

#### Indicator evaluation

The health service indicators from the DHIS were evaluated for suitability to the framework based on their current monitoring purposes and recommendations from existing guidelines. These guidelines included:

- Annual performance plans of the Department of Health in SA.
- Guidelines for maternity care in South Africa.
- The strategic objectives of the global network to improve Quality, Equity and Dignity in maternal, newborn and child health [11].
- Resources exploring the WHO Quality of Care Framework for maternal and new-born health [12, 13].
- Global Review of Key Interventions related to reproductive, maternal, newborn and child health [14].
- Guidelines for positive birth experience with a focus on monitoring Intrapartum care [15].
- Quality of care at primary (Ideal Clinic Realization and Maintenance Program) and hospital (National Core Standards) level in SA [16, 17].

For social determinants of health, we relied on literature focusing on the relationship between interventions and maternal health outcomes. We also relied on recommendations by the WHO and Commission on Social Determinants of Health [18], conceptual framework of the social determinants of health [19], and frameworks for practice at country level [20]. The evaluation of indicators also revealed outstanding gaps in measuring interventions on the framework, which we describe in this study.

### Results

#### Indicator set

**Fig. 2:**
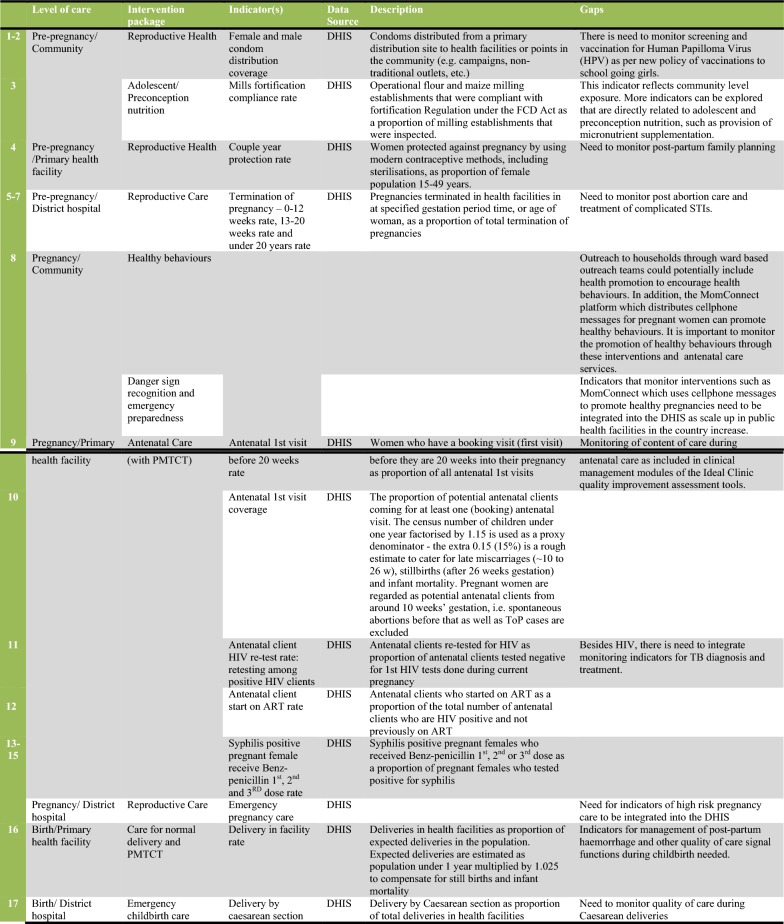

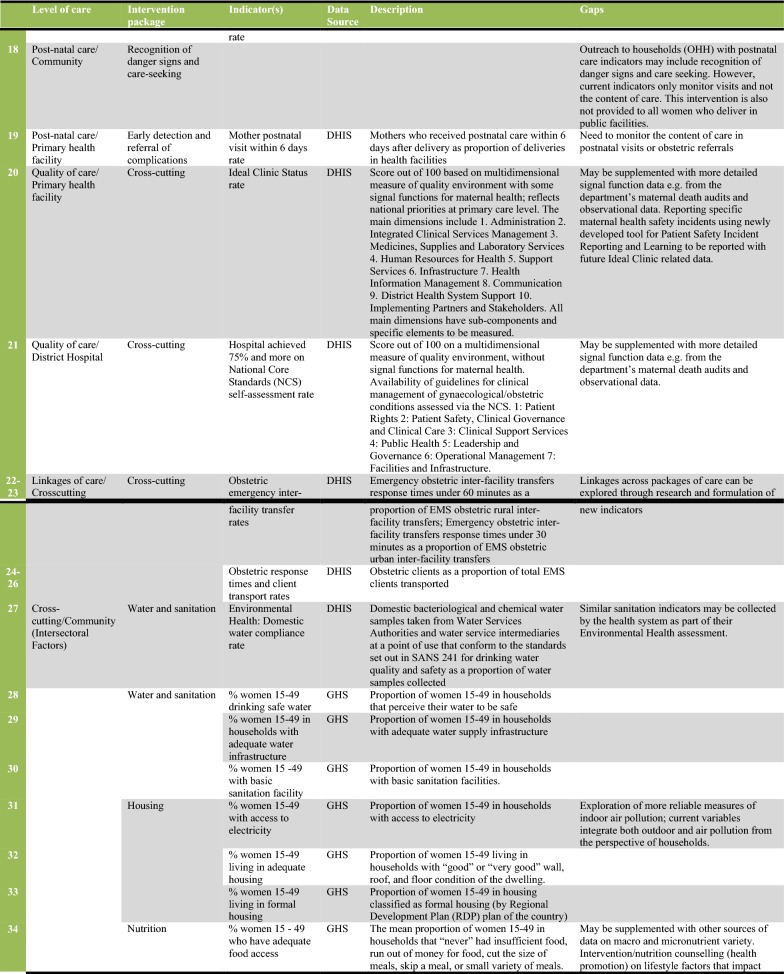

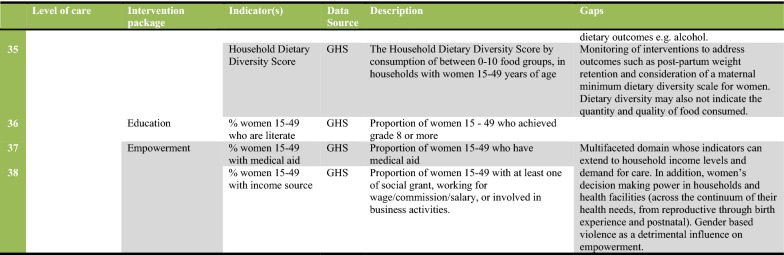
Description of indicators and gaps in monitoring interventions along the continuum of care for maternal health in South Africa. The indicator tool was developed to summarize available indicators, their source, and the data gaps that were observed in the study of the continuum of care for maternal health in South Africa. The levels of care and intervention package columns are based on the continuum of care framework developed by health system actors, and presented in Fig. 1 of this study, and indicators are grouped together to make the figure more concise (e.g. indicators 5–7 represents three indicators within the reproductive care package). The detailed definition and numerator and denominators of each indicator can be found in the metadata of the relevant data sources as specified in the Figure. The description of indicators gives a general guidance of the measures involved. Data gaps are also summaries from literature, global and national guidelines as specified in the manuscript

In Fig. 2 we present a set of 38 indicators that were extracted and evaluated from the DHIS and GHS (27 indicators from the health system and 11 for the intersectoral factors). The figure also describes measurement gaps per intervention package of the CoC. As Fig. 2 shows, indicators are available for most of the intervention packages on the CoC framework. The exceptions were danger sign recognition and emergency preparedness, healthy behaviour promotion and indicators for emergency pregnancy care. The lack of indicators demonstrates unavailable services and/or poor monitoring by the health system. Sometimes indicators are available that do not directly measure maternal health outcomes. Figure 2 shows proxies such as food fortification compliance rates (Indicator 3) used by the health system at community level. Other proxies include Ideal Clinic status (Indicator 20) and national core standards (Indicator 21), which are summary measures of quality of care at facility level. Where only proxies are available, we recommend health information system improvements to measure and integrate measures that are more directly related to maternal health outcomes.

Even where indicators are available, measurement improvements can be made in order to monitor each intervention package comprehensively. Each intervention package consists of more than one intervention, as shown in Fig. 1. For example, while Reproductive Care at district hospital may include timely termination of pregnancy indicators, there is a gap in monitoring post-abortion care and treatment of complicated STIs as part of the package. Thus, more research is needed to assess the extent to which the health system provides services within each intervention package of the CoC. New health system interventions, such as Human Papillomavirus (HPV) vaccinations for school going girls and health promotion through mobile phones (MomConnect program), should be monitored through the DHIS. This will improve the comprehensiveness of the data set and ease of monitoring the CoC in the health system.

Quality of care was an under-measured aspect of interventions such as antenatal care visits, normal and Caesarean deliveries, and postnatal visits. There is a need for intervention specific qualities of care indicators, as exemplified by retest rates for HIV positive clients during antenatal care (Indicator 11, Fig. 2). The health system runs parallel quality of care systems for maternal health, particularly the confidential maternal mortality audits [21]. We recommend the establishment of routine measures from these sources for integration into the DHIS. Routine quality of care monitoring should also include reporting of safety incidents and experience of care surveys disaggregated by population groups.

While indicators are available for the intersectoral factors in the framework, we observed gaps in monitoring indoor air pollution, maternal nutrition counselling, and women’s empowerment for decision making and demand for healthcare. Like health system interventions, each intersectoral factor could be measured by more than one indicator. For instance, in the water and sanitation intervention package, the GHS had variables that could assess safety of water, infrastructure and basic sanitation (Indicators 28–30). While educational achievement indicators may be straightforward, factors such as empowerment and nutrition are more multifaceted. Thus, a variety of indicators can be isolated for their measurement, depending on data availability.

In summary, Fig. 2 is the indicator tool which provides a description of available indicators and gaps that need to be addressed to monitor the CoC for maternal health. The gaps identified should not preclude use of the tool to assess the nature and extent of provision of services along the CoC for maternal health in future studies. The improvement and validation of indicators in maternal health should be a continuous process, tied to evolving policies and information system improvements [22].

#### Adequacy assessment

**Fig. 3:**
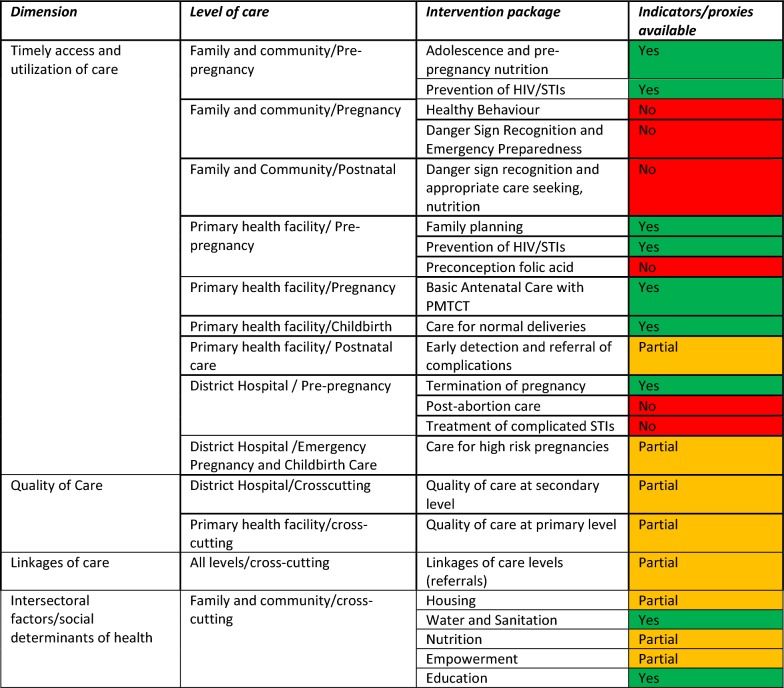
Assessment of availability of indicators over dimensions and domains of the continuum of care for maternal health in South Africa. The dimensions of the continuum of care are defined according to the adequacy construct developed in a previous study [5]. The level of care and intervention packages contain indicators found in Fig. 2 and are based on the continuum of care framework in Fig. 1. We assign “partial” (orange) measurement if indicators are available but there are measurement gaps identified. When assigned “no” (red) when no indicators or proxies were identified from the data sources. And we assigned “yes” (green) if, according to literature and existing guidelines, the indicators available to measure the intervention package are considered adequate

In Fig. 3 indicators are grouped according to adequacy dimensions, and the information in Fig. 2 used to subjectively assess the level to which intervention packages can be measured by available indicators. We assign “partial” (orange) measurement if indicators are available but there are measurement gaps identified. When assigned “no” (red) if no indicators or proxies were identified from the data sources. And we assigned “yes” (green) if, according to literature and existing guidelines, there are indicators available to measure the intervention package. Availability of indicators for an intervention package does not preclude future rigorous validation processes and iterations; this is a normal process within the health information system that is encouraged.

All dimensions of the CoC can be measured by current indicators, although gaps remain within specific intervention packages. Data gaps were most prevalent in the care access and utilization dimension, where 40% (6/15) of intervention packages had no indicators available. Dimensions of quality and linkages of care can only be partially measured; while only 40% (2/5) of social determinants of health domains have available indicators. In general, the GHS and the DHIS provide indicator data for measurement and monitoring of the majority (74%) of CoC intervention packages (17/23) as defined by the framework in Fig. 1.

## Discussion

This study developed and assessed the indicator tool for the continuum of care framework for maternal health in South Africa. This process can be applied to newborn and child health indicators within the framework, using relevant data sources. These processes contribute to the operationalization of the framework, in order to fulfil health system goals in comprehensive monitoring and evaluation of maternal health [23]. Our study also advances the application of the adequacy approach to assess the multi-dimensionality of the available indicators. The adequacy approach complements the framework developed by health system actors by integrating quality of care measures. The CoC has been criticized for under-emphasizing quality of care [24]. For instance, there is still a gap in monitoring quality of care signal functions for maternal health through the DHIS. Data from many programs in the health system are collected separately and only later incorporated into the DHIS [25]. We recommend future research for assessing feasibility of integration of quality of care and service programs data into the routine monitoring and evaluation systems.

Interventions that signify “linkages of care” were also not well defined prior to our study. For that purpose, we proposed the use of indicators for patient transport from community to facility and in-between facilities. Transport facilitates referrals between different levels of care, and an important determinant of maternal mortality in SA [21]. Referrals encompass not only transport but also matching skills to patient needs and managing congestion in facilities [21]. Thus, more research is needed to identify indicators for monitoring human resources and patient management factors in facilities that can contribute to the framework. Our study identified a gap in linkages between one intervention package and another, which is also an important determinant of maternal health outcomes [26, 27]. The CoC framework improves on the country’s strategic plan because it includes more social determinants than water and sanitation [23]. Other frameworks propose even more social determinants, such as occupation, social class, race and ethnicity, social environment and psychosocial circumstances, and behavioural factors [19]. In this study we focused on the domains specified by the framework and recommend future research to explore feasibility of additional indicators.

In conclusion, this study proposed a multidimensional, comprehensive indicator set that can be used to assess the continuum of maternal health care in public health research and practice. The indicator set integrates the under-specified aspects of the framework, such as quality of care and broader social determinants of health, thus improving its potential use from a multisectoral perspective.

## Limitations

The indicators used are only applicable to the South African context, but the adequacy model can be used by researchers from other LMICs to guide a multidimensional analysis of information in their context. We identified and assessed indicators only for the intervention packages outlined in the CoC framework and the dimensions proposed through the adequacy model. We recommend on-going research to refine the framework and indicators suitable for maternal health CoC.

## Data Availability

The datasets generated and/or analysed for the General Household Survey during the current study are available in the DataFirst repository, [https://www.datafirst.uct.ac.za/dataportal/index.php/catalog/central] [10]. The datasets generated and/or analysed for the District Health Information System during the current study are available in the National Department of Health Data Dictionary repository, [https://dd.dhmis.org/] [7].

## Abbreviations

CoC: Continuum of Care
DHIS: District Health Information Systems
GHS: General Household Survey
HPV: Human Papillomavirus
LMIC: Low- and Middle-Income Countries
NIDS: National Indicator Data Set
SA: South Africa
STIs: Sexually Transmitted Illnesses
WHO: World Health Organization

## References

[1] Kerber KJ, de Graft-Johnson JE, Bhutta ZA, Okong P, Starrs A, Lawn JE (2007). Continuum of care for maternal, newborn, and child health: from slogan to service delivery. Lancet.

[2] Bradshaw D (2008). Every death counts: saving the lives of mothers, babies and children in South Africa.

[3] Department of Health. Strategic plan for maternal, newborn, child and women’s health (MNCWH) and nutrition in South Africa: 2012–2016, Pretoria, South Africa; 2012.

[4] Department of Health. Integrated Clinical Services Management, Pretoria; 2015.

[5] Mothupi M, Knight L, Tabana H (2018). Measurement approaches in continuum of care for maternal health: a critical interpretive synthesis of evidence from LMICs and its implications for the South African context. BMC Health Serv Res..

[6] Department of Health. District health management information system (DHMIS) policy, Pretoria; 2011.

[7] Department of Health. Data File: NIDS Integrated. *The NDOH Data Dictionary*, 2019. https://dd.dhmis.org/orgunits.html?file=NIDSIntegrated&source=nids&ver=22b8. Accessed 09 Sept 2020.

[8] Department of Planning Monitoring and Evaluation. Development indicators 2011, Pretoria; 2011.

[9] Statistics South Africa. General household survey: selected development indicators 2016, Pretoria; 2017.

[10] Statistics South Africa. Open Data Portal, *DataFirst*, 2019. https://www.datafirst.uct.ac.za/dataportal/index.php/catalog/central. Accessed 09 Jan 2020.

[11] WHO (2018). Quality, equity, dignity: the network to improve quality of care for maternal, newborn and child health.

[12] Brizuela V, Leslie HH, Sharma J, Langer A, Tunçalp Ö (2019). “Measuring quality of care for all women and newborns: how do we know if we are doing it right ? A review of facility assessment tools. Lancet Global Health..

[13] Tunçalp Ӧ (2015). Quality of care for pregnant women and newborns-the WHO vision. BJOG An Int J Obstet Gynaecol..

[14] The Partnership For Maternal Newborn & Child Health (2011). A global review of key interventions related to reproductive, maternal, newborn and child health.

[15] WHO (2018). Intrapartum care for a positive childbirth experience.

[16] Department of Health. Ideal Clinic Definitions, Components and Checklists, Pretoria, 2018.

[17] Department of Health. Towards Quality Care for Patients. National Core Standards for Health Establishments in South Africa, Pretoria, 2011.

[18] Commission on Social Determinants of Health. Closing the gap in a generation: health equity through action on the social determinants of health. Geneva: World Health Organization; 2008. 10.1016/S0140-6736(08)61690-6 18994664

[19] Solar O, Irwin A (2010). A conceptual framework for action on the social determinants of health:  social determinants of health discussion paper 2 (Policy and Practice).

[20] WHO (2011). Closing the gap: policy into practice for social determinants of health.

[21] Department of Health. Saving mothers 2014–2016: seventh triennial report on confidential enquiries into maternal deaths in South Africa: executive summary, Pretoria; 2016.

[22] Benova L, Moller A, Moran AC (2019). ‘What gets measured better gets done better’: the landscape of validation of global maternal and newborn health indicators through key informant interviews. PLoS ONE.

[23] Department of Health, Strategic plan for maternal, newborn, child and women’s health (MNCWH) and nutrition in South Africa: 2012–2016, Pretoria; 2012.

[24] Graham WJ, Varghese B (2012). Quality, quality, quality: gaps in the continuum of care. Lancet.

[25] Wright G, O’Mahony D, Cilliers L (2017). Electronic health information systems for public health care in South Africa: a review of current operational systems. J Health Inform Africa.

[26] Pattinson RC (2007). Report to UNICEF on the scaling-up of the BANC quality improvement programme in two sub-districts per province in South Africa.

[27] Kikuchi K (2015). Effective linkages of continuum of care for improving neonatal, perinatal, and maternal mortality: a systematic review and meta-analysis. PLoS ONE.

